# In vitro assessment of some sperm function following exposure to levonorgestrel in human fallopian tubes

**DOI:** 10.1186/1477-7827-10-8

**Published:** 2012-01-30

**Authors:** Alexia Hermanny, M Valeria Bahamondes, Francisco Fazano, Nadia M Marchi, Maria Elena Ortiz, Maria Heloisa RR Genghini, Horacio B Croxatto, Luis Bahamondes

**Affiliations:** 1 Human Reproduction Unit, Department of Obstetrics and Gynecology, School of Medicine, University of Campinas (UNICAMP), 13084-971 Campinas, SP, Brazil; 2 Instituto Chileno de Medicina Reproductiva (ICMER), Santiago, Chile; 3 Departamento de Biología, Facultad de Química y Biología, Universidad de Santiago de Chile, Santiago, Chile

**Keywords:** Emergency contraception, Levonorgestrel, Acrosome reaction, Fallopian tubes, Human spermatozoa

## Abstract

**Background:**

The mechanism of action of levonorgestrel (LNG) as emergency contraception (EC) remains a subject of debate and its effect on sperm function has been only partially explained. The aim of this study was to assess whether LNG at a similar dose to those found in serum following oral intake for EC could affect spermatozoa when exposed to human fallopian tubes in vitro.

**Methods:**

Fifteen mini-laparotomies were performed, the side on which ovulation occurred was recorded, and both tubes were removed and perfused with a suspension containing 1 × 10(6) motile spermatozoa, with or without LNG. Following 4-hour incubation, the tubes were sectioned to separate the isthmus and the ampulla. Each segment was flushed and the material was evaluated to quantify the number of motile sperm, the number of spermatozoa adhering to the oviductal epithelium and the acrosome reaction (AR) rate.

**Results:**

The addition of LNG did not significantly alter the number of recovered motile spermatozoa either at the isthmus or at the ampulla, nor did it have any effect on the number of recovered spermatozoa adhered to the human tubal epithelium. Furthermore, LNG did not affect the AR rate. No significant differences were found even when the side on which ovulation occurred was taken into account.

**Conclusions:**

In a similar dose to that observed in serum following oral intake for EC, LNG had no effect on the number of motile spermatozoa recovered from the human fallopian tubes in vitro, on their adhesion to the tubal epithelium, distribution or AR rate. The possible effect of LNG as EC on sperm function remains poorly understood.

## Background

Levonorgestrel (LNG) is a progestin used in emergency contraception (EC). The currently recommended LNG dose consists of two 0.75 mg pills taken 12 h apart or one dose of 1.5 mg up to 72 h after unprotected sexual intercourse [1,2]. Despite the disseminated use of LNG as EC worldwide, its mechanism of action remains under debate and may involve several mechanisms that are dependent on the time of administration in relation to sexual intercourse and on the phase of the menstrual cycle in which it is taken [3,4].

One of the proposed mechanisms of action concerns the effect of LNG on spermatozoa and their functions [5-8]. However, this effect is still poorly understood. Progesterone (P) promotes changes in aspects of sperm function related to fertilization such as capacitation, sperm hyperactivation and acrosome reaction (AR) [9-15]. Although LNG exerts a weak agonistic effect on P sperm receptors, high LNG concentrations (200-800 ng/mL) have been shown to be capable of inducing AR in human spermatozoa in vitro [8]. However, when the spermatozoa were exposed to LNG concentrations similar to those observed in serum after intake for EC [16], no effect was observed [17]. Additionally, when human spermatozoa were treated in vitro with LNG at doses of 1 ng/mL, 10 ng/mL and 100 ng/mL, representing lower, similar and higher levels than those observed in serum after LNG intake for EC, motility was found to be impaired with the 10 ng/mL dose; however, no effect was observed on the AR [7]. Otherwise, administration of 1.5 mg of LNG to sterilized women at different moments following coitus failed to have any effect on the quality of cervical mucus, sperm penetration to the uterine cavity or AR [18].

After ejaculation, mammalian spermatozoa are unable to fertilize the oocyte and this capacity is subsequently acquired as a consequence of a series of physiological and functional changes called capacitation. Sperm capacitation occurs during sperm migration within the female genital tract [19]; however, it has been noted that adhesion of spermatozoa to the tubal epithelium constitutes an important condition for in vivo fertilization in several species [20]. The interaction between spermatozoa and the endosalpinx offers some protection to spermatozoa and in vitro sperm motility has been found to be longer when spermatozoa are incubated with tubal epithelium [21]. Spermatozoa undergo two forms of change in preparation for fertilization: capacitation and hyperactivation. Capacitation includes changes at the plasma membrane including the protein layer and several lipids that prepare the cells for the AR and fertilization [22]. Those spermatozoa that complete AR precociously are unable to penetrate the zona pellucida (ZP) because they lose enzymatic acrosome content [10,23].

The effect of LNG as EC on sperm function is still poorly understood and to the best of our knowledge, this effect has not been tested at the site of fertilization. Therefore, the objective of this study was to assess whether LNG, at a similar dose to those observed in serum following oral intake for EC, could affect spermatozoa exposed to human fallopian tubes under in vitro conditions.

## Methods

The study was conducted at the Human Reproduction Unit, Department of Obstetrics and Gynecology, School of Medicine, University of Campinas (UNICAMP), Campinas, Brazil. All the women and semen donors gave their written informed consent and the study protocol was approved by the institution's internal review board. Women of 25-41 years of age were invited to participate in the study. The admission criteria consisted of: women who had requested surgical sterilization and for whom the abdominal route of surgery was indicated, with regular menstrual periods (25-35 day intervals), no known tubal diseases, not having used hormonal or intrauterine contraceptives during the cycle of the experiment, not having used any other form of hormone therapy, and not having been breast feeding or pregnant in the 6 months preceding the study.

### Experiments

Women were instructed to use condoms as their method of contraception from the first day of the cycle until the day of surgery in order to avoid unplanned pregnancy. Follicular development was monitored daily in all participants from the 8^th ^day of the menstrual cycle by ultrasound with a 5.0 MHz vaginal probe (Justavision 400, Toshiba, Toshigi-Ken, Japan). When the dominant follicle reached a diameter of 14-17 mm, surgical sterilization was scheduled for the following day. All in vitro experiments were performed prior to ovulation to avoid the effect of the P milieu on AR and any possible confounding effect of P on sperm adhesion to the tubal epithelium. To confirm the follicular phase of the menstrual cycle, a blood sample was drawn on the day of surgery and the serum was separated and stored at -20°C until analysis. Serum P levels < 3 ng/mL were considered as confirmation of the follicular phase of the cycle [24]. P levels were measured in duplicate using a commercial electrochemiluminescence immunoassay (Roche Diagnostics GmbH, Mannheim, Germany) with a measurement range of 0.030-60.0 ng/ml, an inter-assay coefficient of variation (CV) of 2.4% and an intra-assay CV of 2.7%.

All the women were anesthetized and mini-laparotomy was then performed. Both fallopian tubes were removed gently from the proximal portion. The side on which the dominant follicle was present was identified and recorded. The tubes were placed in two separate Petri dishes with HEPES-buffered modified Human Tubal Fluid medium (HTF-HEPES; Irvine Scientific, Santa Ana, CA, USA) and transferred immediately to the laboratory. The excess tissue (mesosalpinx) was removed.

### Semen samples

Fifteen semen samples were obtained from healthy donors with normal sperm according to the analysis criteria defined in the World Health Organization manual [25]. Semen was collected by masturbation in sterile plastic jars after 3 days of sexual abstinence. After evaluation, the sample was separated into two tubes of equal volume. Motile spermatozoa of each fraction were selected by a swim-up technique in which 1 mL of HTF-HEPES medium supplemented with 35 mg/mL of bovine serum albumin (BSA), 1 nM of estradiol (E_2_) and 25 nM of LNG (0.1% solution obtained from a stock solution containing 25 μM of LNG in ethanol) were used in one tube, while the same final concentration of ethanol present in the LNG-treated sample was added to the other tube. After 1 h of incubation, the supernatant with the motile spermatozoa was carefully removed and an aliquot was placed into a Makler counting chamber (Sefi Medical Instruments, Haifa, Israel), warmed to 37°C and examined under a microscope. After evaluation, each fraction was re-suspended in the same medium to achieve a sperm concentration of 10 × 10^6 ^motile spermatozoa/mL. A sample of each sperm suspension was taken to evaluate the AR as described below.

### Fallopian tube perfusion

Using a blunt needle, one of the fallopian tubes was perfused from the proximal to the distal portion with 100 μL of a sperm suspension containing 1 × 10^6 ^motile spermatozoa in the medium described above, including LNG, while in the other tube the LNG in the sperm suspension was substituted for the same concentration of ethanol present in the LNG-treated sample. The fallopian tube corresponding to the ovary with the dominant follicle received the suspension containing LNG, while the other tube received the suspension containing only the vehicle. Therefore, the tube that received LNG (either the right or left tube) varied in the women according to the presence of the dominant follicle in the ipsilateral ovary. Perfusion was carried out very slowly to avoid spilling any of the solution outside the tube. The procedure was performed outside the culture medium, over a glass, under controlled temperature conditions. After perfusion with the sperm suspension, the tubes were incubated separately at 37°C for 4 h in a Petri dish containing the same medium used in the sperm suspension to allow capacitation to occur.

### Fallopian tube processing

After incubation, the tubes were sectioned to separate the isthmus and the ampulla, and each segment was flushed twice, first with 5 ml of HTF-HEPES medium and then with an equal volume of phosphate-buffered saline (PBS; GIBCO, BRL, Life Technologies, Inc, Grand island, NY, USA) medium containing 0.5% Triton-X100 (Sigma-Aldrich, St Louis, Missouri, USA), a nonionic surfactant, in order to remove the spermatozoa adhering to the oviductal epithelium [26,27]. The material flushed from the tubal segments was centrifuged and the pellets were re-suspended in 100 μL of PBS. The material from the first flushing with HTF-HEPES medium was evaluated to assess the number of motile sperm and the AR rate. The material obtained from flushing with Triton-X100 was evaluated to asses only the number of recovered spermatozoa since all the sperm cells died when in contact with the Triton-X100 medium. The culture medium from the Petri dish in which the tubes were immersed during incubation was transferred to test tubes and centrifuged and the pellets were also diluted in 100 μL of PBS for further evaluation of the number of motile sperm and AR rate.

The samples obtained following flushing with Triton were evaluated in a Neubauer chamber to verify the number of spermatozoa recovered in each segment. The other samples were divided to allow 50 μL to be used for counting the number of motile spermatozoa in a Makler counting chamber and another 50 μL to be used for analysis using Hoechst stain. A volume of 50 μL of 1 μg/mL Hoechst 33258 (bisbenzimide; Sigma Chemical Co; St. Louis USA, B2883) was added to the 50 μL aliquot of the washing medium containing the recovered spermatozoa. The mixture was incubated for 5 min at 37°C, washed twice with PBS to remove the excess stain and then centrifuged. The pellets were re-suspended in 50 μL of PBS. Two slides from each washing were prepared and allowed to dry in a dark environment at room temperature.

### Assessment of AR status

The fluorescent probe fluorescein isothiocyanate-labelled *Pisum sativum *agglutinin (FITC-PSA; Sigma-Aldrich) was used to evaluate AR status. After being left to dry, the slides prepared from the spermatozoa suspension stained with Hoechst were immersed in cool absolute methanol at -20°C for 30 s. Next, the slides were stained by immersing them in FITC-PSA at a concentration of 40 μg/mL in PBS for 30 min, protected from light at room temperature. After incubation, the slides were washed in PBS and stored in the dark until analysis of AR and vitality. The slides were evaluated using a fluorescent microscope (Zeiss, Axioplan II, Jena, Germany) equipped with a specific filter for the FITC-PSA method (494-blue excitation; 520 emission; 510-514 barrier) and with a filter for the Hoechst 33258 stain (343-UV excitation; 480 emission; 400 barrier). Two hundred cells were evaluated in randomly selected fields.

The spermatozoa that were considered acrosome-reacted were those with the following patterns: (i) patchy fluorescence in the acrosome region (partially acrosome reacted) and (ii) fluorescence of the equatorial band only (acrosome reacted) [28]. The AR rate was defined as the difference between the rate observed in the perfusion material prior to incubation and the rate observed in sperm suspension obtained after the segment was flushed.

### Statistical analysis

The SAS statistical software program, version 9.2, was used to analyze the data. The mean number of recovered spermatozoa and the AR rate with and without LNG were compared using the Mann-Whitney test. The level of significance was established at p < 0.05 and all values are shown as means ± standard error of the mean (SEM).

## Results

Fifteen experiments were conducted. However, two women had P levels > 3 ng/mL on the day of the experiment and were excluded from the analysis to avoid the effect of P on the AR. Most of the spermatozoa recovered after the first tubal flushing was motile. The number of motile spermatozoa recovered in the isthmus was similar, irrespective of whether LNG had been added or not. Nevertheless the number of recovered motile spermatozoa was almost 10 times greater at the ampulla than at the isthmus, although there was also no difference with respect to whether LNG had been added to the medium or not. When the number of spermatozoa recovered after flushing with Triton was considered, the addition of LNG was found to have had no effect and the numbers were fairly similar, although the number of spermatozoa recovered from the ampulla was ~ 5 times greater than the number recovered from the isthmus (Table 1). There were no significant differences between the number of recovered motile spermatozoa in the medium to which LNG had been added and the medium to which LNG had not been added. The values found in the medium were almost five times greater than those observed when the number of motile spermatozoa at the isthmus and the number at the ampulla are taken together. Of the samples obtained after flushing with Triton, two samples had to be excluded from each segment because of technical problems that occurred during recovery of the sperm cells (Tables 1 and 2).

**Table 1:** Total number of recovered spermatozoa (× 10^3^) according to the segment of the Fallopian tube and treatment

	LNG in the medium	N	Sperm count (Mean ± SEM)	Range	*p*-value
Isthmus*					0.66

	Yes	13	4.6 ± 1.7	0-20	

	No	13	3.8 ± 1.7	0-15	

Isthmus with Triton**					0.23

	Yes	11	0.5 ± 0.2	0-2	

	No	11	1.6 ± 0.7	0-7	

Ampulla*					0.54

	Yes	13	38.9 ± 11.8	0-130	

	No	13	33.5 ± 12.1	0-140	

Ampulla with Triton**					0.71

	Yes	11	6.8 ± 2.8	0-26	

	No	11	5.2 ± 3.0	0-33	

Medium*					0.30

	Yes	13	181.9 ± 87.8	0-1,200	

	No	13	217.7 ± 85.4	30-1,200	

*Motile spermatozoa recovered

**The spermatozoa from the flushing with Triton were considered adhered to the Fallopian epithelium.

Medium: refers to the medium used in the experiments in which the tubes were immersed during incubation.

**Table 2:** Total number of recovered spermatozoa (× 10^3^) according to the side with or without ovarian follicle and according to the segment of the tube and treatment

	LNG in the medium	N	Sperm count (Mean ± SEM)	Range	*p*-value
**With ovarian follicle**					

*Isthmus*					0.26

	Yes	6	2.5 ± 1.7	0-10	

	No	7	7.1 ± 2.6	0-15	

Isthmus with Triton*					0.14

	Yes	5	0.2 ± 0.2	0-1	

	No	6	2.3 ± 1.1	0-7	

*Ampulla*					0.40

	Yes	6	51.7 ± 23.7	0-130	

	No	7	22.9 ± 10.4	0-80	

Ampulla with Triton*					1.00

	Yes	5	8.8 ± 5.5	0-26	

	No	6	3.8 ± 2.0	0-12	

*Medium*					

	Yes	6	67.5 ± 31.2	0-190	0.12

	No	7	300.7 ± 152.6	30-1200	

**Without ovarian follicle**					

*Isthmus*					0.07

	Yes	7	6.4 ± 2.8	0-20	

	No	6	0.0	0	

Isthmus with Triton*					1.00

	Yes	6	0.7 ± 0.3	0-2	

	No	5	0.8 ± 0.6	0-3	

*Ampulla*					1.00

	Yes	7	27.9 ± 8.7	0-65	

	No	6	45.8 ± 23.6	0-140	

Ampulla with Triton*					0.52

	Yes	6	5.2 ± 2.8	0-18	

	No	5	6.8 ± 6.6	0-33	

*Medium*					0.68

	Yes	7	280.0 ± 156.4	30-1200	

	No	6	120.8 ± 43.3	30-285	

*The spermatozoa from the flushing with Triton were considered adhered to the Fallopian epithelium.

Medium: refers to the medium used in the experiments in which the tubes were immersed during incubation.

The number of motile spermatozoa recovered was similar both in the tubal isthmus and in the ampulla irrespective of whether LNG had been added or not. There was no difference in the number of motile spermatozoa recovered from the tubes irrespective of whether the tube was from the side with the dominant follicle or from the opposite side. In addition, there were no statistically significant differences in the number of spermatozoa recovered after flushing with Triton. Nevertheless, the number of spermatozoa was greater in the ampulla than in the isthmus on both sides irrespective of whether LNG had been added or not (Table 2).

After preparing the slides for evaluation of the AR, some were found to contain numerous epithelial cells and red and white blood cells, making it almost impossible to evaluate the AR rate accurately. For this reason, some of the evaluations were excluded from the analysis (Tables 3 and 4). The AR rate was also similar in the spermatozoa recovered from the isthmus and in those recovered from the ampulla irrespective of whether the cells had been treated or not with LNG. In addition, there were no significant differences between the AR rates in the medium irrespective of whether it had been treated with LNG or not (Table 3). No statistically significant differences were found in AR in the spermatozoa from the isthmus compared to those from the ampulla, in the medium to which LNG had been added compared to the medium without LNG, or on the side of the dominant ovarian follicle compared to the opposite side. Furthermore, no statistically significant differences were found in the percentage of AR observed in the spermatozoa from the tubal segments and medium as a function of whether they were obtained from the side with the dominant ovarian follicle or not (Table 4).

**Table 3:** Percentage of spermatozoa with acrosomal reaction according to the segment of the Fallopian tube and treatment

	LNG in the medium	N	Acrosome reacted spermatozoa (%) (Mean ± SEM)	*p*-value
*Isthmus*				0.57

	Yes	9	15.2 ± 3.3	

	No	9	18.7 ± 2.5	

*Ampulla*				0.96

	Yes	9	9.1 ± 1.2	

	No	8	9.4 ± 1.9	

*Medium*				0.28

	Yes	9	5.5 ± 1.9	

	No	9	3.8 ± 2.1	

Medium: refers to the medium used in the experiments in which the tubes were immersed during incubation.

**Table 4:** Percentage of spermatozoa with acrosomal reaction according to the side with or without ovarian follicle and according to the segment of the fallopian tube and treatment

	LNG in the medium	N	Acrosome reacted spermatozoa (%) (Mean ± SEM)	*p*-value
**With ovarian follicle**				

*Isthmus*				0.15

	Yes	4	7.9 ± 3.2	

	No	5	19.9 ± 3.9	

*Ampulla*				0.35

	Yes	4	7.5 ± 1.9	

	No	5	11.2 ± 2.5	

*Medium*				0.77

	Yes	3	5.5 ± 3.1	

	No	5	2.8 ± 3.8	

**Without ovarian follicle**				

*Isthmus*				0.42

	Yes	5	21.0 ± 3.8	

	No	4	17.1 ± 3.4	

*Ampulla*				0.40

	Yes	5	10.4 ± 1.5	

	No	3	6.3 ± 2.6	

*Medium*				0.54

	Yes	6	5.5 ± 2.6	

	No	4	5.1 ± 1.5	

Medium: refers to the medium used in the experiments in which the tubes were immersed during incubation.

## Discussion

These results show that the addition of LNG did not significantly affect the number of recovered motile spermatozoa either at the isthmus or at the ampulla nor did it affect the number of recovered spermatozoa adhered to the tubal epithelium. With respect to whether the addition of LNG affected the number of recovered motile spermatozoa, the number of spermatozoa adhered to the tubal epithelium or the AR rate in the flushing medium on the side with the dominant ovarian follicle or on the opposite side, no statistically significant differences were found.

However, the principal limitation of this study refers to the design and development of the procedures. The experiments were conducted in vitro and, although the spermatozoa were tested in the tubal environment, conditions differed from those encountered in vivo. The doses of LNG used for flushing the tubes are similar to serum levels found after oral intake as EC and possibly higher than those found in oviductal fluid. In fact, as previously reported by our group [18], the LNG concentration in uterine flushing after oral intake was less than 2% of that found in serum [16] and concentrations at the tubal lumen are probably similar. Furthermore, these findings do not permit us to affirm that the administration of LNG as EC in vivo affects the oviductal microenvironment, impairing sperm function. In fact, the best experiment may be for women to take a dose of LNG as EC prior to having their tubes removed to evaluate whether or not LNG exerts any effect on oviductal P receptors and whether it is able to modify the oviductal microenvironment and its interaction with spermatozoa. The experiments conducted in the present study did not mimic this situation.

Previous in vitro experiments have shown that at a dose of 10 ng/mL, LNG was unable to induce AR [7,17] and also failed to induce any significant changes in the number or AR status of spermatozoa recovered from the uterus after administration of 1.5 mg of LNG to sterilized women at different times following coitus [18]. However, to the best of our knowledge, no studies have been conducted in which the effect of LNG on spermatozoa was evaluated at the site of fertilization. With the intention of contributing to the understanding of the mechanism of action involved in the contraceptive effect of LNG as an EC, the objective of the present study was to determine whether the drug was able to affect the sperm count, the number of spermatozoa adhered to the tubal epithelium, their distribution and AR in human fallopian tubes in vitro.

The methodology used in this study could not be better. Initially, an attempt was made to recover spermatozoa from tubes obtained at the time of sterilization from women who had had unprotected coitus and received LNG as EC. However, the number of spermatozoa recovered after perfusion of the tubes was extremely low and did not permit any further analysis to be made, a result that had also been reported previously using the same technique [29]. Consequently, it was decided to work in vitro, perfusing the tubes with a predetermined number of spermatozoa and evaluating sperm function after incubation.

It is well known that women are fertile for six days in the menstrual cycle: the five days preceding ovulation and the day of ovulation itself [30]. Recently Noé et al. [31] observed that despite the evidence of follicular rupture in women who had received LNG as EC prior to follicular rupture, no pregnancies occurred among those women, suggesting that mechanisms other than ovulation suppression prevent pregnancy when LNG is administered as EC to exposed women.

Sperm migration to the oviducts and adhesion to the tubal epithelium, a well-known mechanism of achieving fertilization, are controlled by sex steroids. In rats, E_2 _has been reported to facilitate sperm migration from the uterus to the oviducts and P modulated this effect [27]. In addition, the interaction between E_2 _and P stimulated sperm adhesion to the tubal epithelium. Although no different sperm reservoir has been found in human tubes, a functional reservoir has been described and in vitro, spermatozoa were observed adhered to the epithelium of the isthmus, albeit in an intermittent manner [32,33]. Ortiz et al. [34] observed in vitro that after 3 h of incubation, the number of spermatozoa adhered per 0.1 mm^2 ^of tubal explants decreased both at the isthmus and at the ampulla when LNG was added. However, in the present study, a different methodology was used in that the tubal segments were incubated for 4 h and flushed with a nonionic surfactant (Triton-X100). In addition, the number of spermatozoa obtained in each segment of the tube was recorded separately. LNG had no effect on the number of adhered spermatozoa recovered or on their distribution along the fallopian tubes.

When ovulation is about to take place, spermatozoa undergo capacitation and hyperactivation and are able to progress to the tubal ampulla. In addition, P modulates many aspects of sperm function and mimics the AR-inducing properties of the follicular fluid almost perfectly, while the effects of P on sperm function are mediated by receptors located in the plasma membrane, [35] defined as nongenomic, since they are rapid and do not involve transcriptional processes [36]. It is well known that response is absolutely dependent on the presence of extracellular bicarbonate, [37,38] which is present in intraluminal oviductal fluid at levels higher than those found in peripheral blood [39].

There are two classes of nongenomic P receptors in the human spermatozoa. One has an elevated affinity (at nanomolar concentrations) and is specific for P, while the other has low affinity (micromolar) and also binds to other hydroxylated P derivatives [36]. When P activates human spermatozoa, an increase is seen in intracellular free calcium that may trigger the process of AR [36,40]. Progestogens such as LNG are unable to mimic the effect of P in elevating intracellular calcium and appear to act as very weak agonists in nongenomic receptors compared to their potent effect on genomic receptors [40].

With respect to the effect of P on the AR process, some studies were carried out to evaluate whether LNG was able to affect sperm function as P does. A direct relationship was found between an increase in the AR rate and higher LNG concentrations, suggesting that at higher LNG concentrations (200-800 ng/mL) the nongenomic P receptors on the surface of spermatozoa are able to recognize the progestogen molecule and exert their effect [8]. Nevertheless, the studies that used a similar dose of LNG to that observed in serum following oral intake for EC failed to show any effect of LNG on AR in spermatozoa in vitro [7,17] or in spermatozoa recovered from the uterus [18].

## Conclusions

In conclusion, the hypothesis that LNG, in a similar dose to those found in the serum of women following oral intake for EC, may affect the number of motile spermatozoa, the adhesion of sperm to the tubal epithelium, distribution and the AR of spermatozoa in the fallopian tubes in vitro was not confirmed in the present study. The present findings also failed to show any effect on motile spermatozoa recovered after exposure to the fallopian tubes despite the fact that the doses of LNG used in vitro are probably higher than those reached within the oviduct in vivo.

## Abbreviations

AR: Acrosome reaction; CV: Coefficient of variation; EC: Emergency contraception; E_2_: Estradiol; FITC-PSA: Fluorescein isothiocyanate-labelled *Pisum sativum *agglutinin; HTF-HEPES: HEPES-buffered modified human tubal fluid medium; LNG: Levonorgestrel; P: Progesterone; SEM: Standard error of the mean; ZP: Zona pellucida

## Competing interests

The authors declare that they have no competing interests.

## Authors' contributions

AH, MEO, HBC and LB participated in the design of the study and in developing the research protocol; AH, MVB, FF, NMM, MHRRG, and LB conducted the study at the outpatient clinic, surgical theatre and at the laboratory. All the authors contributed equally in writing the manuscript and in reviewing and revising it. All authors read and approved the final manuscript.
